# Effects of pinacidil on changes to the microenvironment around the incision site, of a skin/muscle incision and retraction, in a rat model of postoperative pain

**DOI:** 10.3892/mmr.2015.3465

**Published:** 2015-03-10

**Authors:** SU CAO, YINBIN QIN, JUNJIE CHEN, SHIREN SHEN

**Affiliations:** Department of Anesthesiology, Affiliated Hospital of Nantong University, Nantong, Jiangsu 226001, P.R. China

**Keywords:** skin/muscle incision and retraction, pinacidil, microenvironment, microvessel density, nerve growth factor, glucose transporter protein-1, C-jun N-terminal kinases

## Abstract

The aim of the present study was to evaluate the influence of the microenvironment around an incision site, on peripheral and central sensitization. The effects of pinacidil activation of ATP-sensitive potassium (KATP) channels prior to skin/muscle incision and retraction (SMIR) surgery were assessed. A total of 24 male Sprague Dawley rats were randomly assigned to four groups: Control, sham (incision operation), SMIR (incision plus retraction 1 h after the skin/muscle incision) and pinacidil (SMIR plus pinacidil). The rats in the pinacidil group were intraperitoneally injected with pinacidil prior to the SMIR procedure. The mechanical withdrawal threshold (MWT) was determined at each time point. The microvessel density (MVD) value was determined by immunohistochemistry, and western blotting was performed to analyze the relative protein expression levels of nerve growth factor (NGF), glucose transporter protein-1 (GLUT1) and C-jun N-terminal kinases. There was a significant reduction in the levels of MVD, GLUT1 and MWT following SMIR surgery as compared with the incision alone, and a significant increase in the NGF protein expression levels. In the SMIR group, the MVD value was significantly increased seven days after surgery, as compared with three days after surgery. Additionally, intraperitoneal administration of pinacidil prior to the SMIR surgery inhibited the SMIR-induced reduction in MWT and MVD and attenuated the SMIR-induced GLUT1 reduction. The results of the present study suggest that the microenvironment around an incision site may affect the development of peripheral and central sensitization. In addition, pinacidil had an inhibitory effect on the formation of the inflammatory microenvironment around the incision site through activation of KATP channels, thereby inhibiting peripheral and central sensitization.

## Introduction

Postoperative pain is a common clinical symptom, predominantly caused by peripheral and central sensitization from the persistent excitement of nociceptors (1). The mechanisms involved in the occurrence and development of postoperative pain remains unclear.

Previous studies have demonstrated that vascular dysfunction and vascular endothelial cells have a role in mechanical pain (2,3); however, few studies have investigated the effects of microvessel density (MVD) on postoperative pain. MVD is generally considered to be a quantitative parameter for vascular endothelial cell function and angiogenesis (4). It has been previously observed that MVD is positively correlated with the expression of glucose transporter protein-1 (GLUT1), which functions in basal metabolism and can enhance glucose utilization (5,6). Furthermore, GLUT1 has been demonstrated as having a crucial role in osteoarthritic pain (7). Nerve growth factor (NGF), a multi-functional nutritional factor, is produced by innervated target organs, and undergoes transportation into nerve terminals (8–10). An overproduction of NGF has been shown to be capable of inducing central sensitization (11,12). In addition, previous studies have shown that the persistent activation of C-jun N-terminal kinases (JNK) in spinal astrocytes, following nerve injury and inflammation, can induce central sensitization (13,14). Furthermore, the activation of ATP-sensitive potassium (KATP) channels has been implicated in mediating anti-nociceptive effects following ventricular, intrathecal or epidural injection of KATP activators in numerous animal models (15,16); however, the direct effects of KATP activators on sensory neurons remains unclear (17).

Previous studies have investigated the anti-nociceptive effects of KATP inhibitors or activators administered peripherally (18,19), yet studies regarding the histopathological changes around the incision site, in response to KATP stimulation, are scarce (16,17). Thus, there remains uncertainty regarding the direct effects of KATP activators on nociceptors.

The present study established a rat model of postoperative pain evoked by skin/muscle incision and retraction (SMIR) surgery (20). The KATP activator pinacidil was intraperitoneally injected prior to surgery. The direct effects of pinacidil on nociceptors around the incision site, were assessed by detecting the mechanical withdrawal thresholds (MWT). The changes to the levels of MVD, as well as the changes to the relative protein expression levels of GLUT1, NGF and spinal JNK were observed both prior to and following the surgery. In addition the effects of the microenvironment surrounding the incision site on mechanical allodynia, following SMIR surgery, and the effects of pinacidil on mechanical allodynia and its mechanisms of action, were determined.

## Materials and methods

### Animal grouping

The present study was approved by the Experimental Animal Protection and Care Committee of Nantong University (Jiangsu, China). A total of 24 male Sprague Dawley rats (weighing 200–250 g) were obtained from the Laboratory Animal Center of Nantong University (Jiangsu, China), and maintained in conventional housing. The rats were randomly assigned to four groups (n=6/group): Control, sham (incision operation), SMIR (incision plus retraction), and pinacidil (SMIR plus pinacidil) groups. The rats in the control group did not receive any treatment. The rats in the sham operation group had an incision made through the skin and muscle. The rats in the SMIR group underwent 1 h retraction after the skin/muscle incision. The rats in the pinacidil group were further divided into three subgroups, and were intraperitoneally injected with either low (10 *μ*g/kg), middle (25 *μ*g/kg), or high-dose pinacidil (50 *μ*g/kg) (Sigma-Aldrich, St Louis, MO, USA; lot number: D9035-250MG) prior to the SMIR procedure.

### Behavioral assessments

All of the rats were adapted to the testing conditions for three days prior to experimentation. To quantify mechanical allodynia, the MWT was determined using von Frey filaments (range 1.4-2.6 g; North Coast Medical Inc., Morgan Hill, CA, USA), as described by previous methods (21). Briefly, each rat was placed in a Plexiglass^®^ box (Nantong Jingxin Optical Glass, Co., Ltd., Nantong, China) with a wire mesh floor. Following habituation for 30 min to the environment, the von Frey filament was pressed perpendicular to the plantar surface of both hind paws and held for ≤4 sec. A positive response was noted if the rats showed paw withdrawal, flinches or licking. If there was no response (negative), the next heavier filament was tested. Each trial was repeated five times. At each 30 sec interval, the 50% threshold was determined using the “up and down” method, as previously described (22); in which if no two consecutive positive responses appeared, a further heavier stimulation was administered; if two consecutive positive responses appeared, a lighter stimulation was administered until the alternation of one positive and one negative response was reached. The alternations with five repetitions were recorded.

### Establishment of the SMIR model

**Figure 1:**
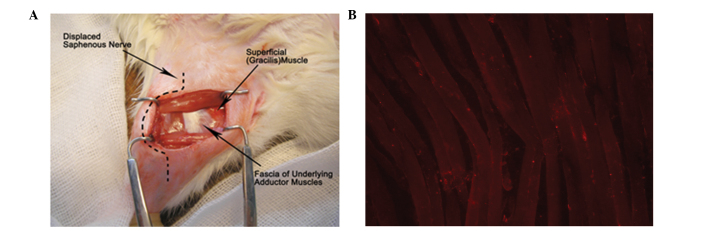
Model of the skin/muscle incision and retraction method used to evoke pain in the rat. (A) Image of the injury site during 1 h retraction period of skin/muscle incision and retraction surgery. (B) Negative control of the microvessel density immunohistochemical staining (magnification, ×200) of the Cy3-conjugated secondary antibody.

The rats were anesthetized with an intraperitoneal injection of nembutal (40 mg/kg; Beijing Propbs Biotechnology Co., Ltd., Beijing, China), and laid in a supine position under sterile conditions. A 1.5–2 cm incision was made in the medial side of the right hind limb ~4 mm medial to the saphenous vein, in order to reveal the thigh muscle. An incision (7–10 mm long) was made in the superficial muscle layer of the thigh. The superficial muscle was then retracted 2 cm by spreading blunt scissors within the muscle incision site. This retraction was maintained for 1 h. During the retraction period, the incision site was covered with gauze and moistened with sterile saline, in order to prevent dehydration of the surgical site. Following the SMIR procedure, the incision was covered with gauze coated with gentamycin (Yantai Justaware Pharmaceutical Co., Ltd., Yantai, China) to prevent infection. The establishment of the injury site during the 1 h retraction period of the SMIR surgery is shown in Fig. 1.

### Western blot analysis

Three days after the SMIR surgery, the rats in each group were anesthetized as described previously. The peripheral muscle and lumbar regions 3–5 of the spinal cord were harvested and homogenized on ice in SDS sample buffer (10 ml/mg tissue), containing a cocktail of proteinase and phosphatase inhibitors (Sigma-Aldrich), using a hand-held pestle. The protease inhibitor cocktail (P2714) contains AEBSF, E-64, bestatin, leupeptin, aprotinin, and sodium EDTA, and the phosphatase cocktail (P5726) contains sodium orthovanadate, sodium molybdate, sodium tartrate, and imidazole. The cell lysates were collected and transferred to a 1.5 ml centrifuge tube. After centrifugation at 10,000 × g for 18 min at 4°C, the protein was extracted, denatured and stored at 4°C, until further use. To determine the protein concentrations, the protein samples were diluted with double- distilled H_2_O five times, and the diluted samples were plated into a 96-well plate. The protein concentration was measured using a bicinchoninic acid kit (Pierce Biotechnology, Inc., Rockford, IL, USA). A Synergy 2 Multi-Mode reader (Biotek Instruments, Inc., Winooski, VT, USA) was used to measure the optical density of each sample, at a wavelength of 562 nm (OD>0.995). The protein concentration of each sample was calculated by referring to a standard curve of the standard reference.

Subsequently, equal amounts (40 *μ*g/lane) of total protein from each sample were separated by 5 and 10% SDS-PAGE (Beyotime Biotech Inc., Nanjing, China) sequentially, and transferred to polyvinylidene difluoride membranes (EMD Millipore, Shanghai, China). The required protein volume per lane was calculated by dividing the total protein amount loaded per lane, by the protein concentration. The membranes were incubated overnight at 4°C with one of the following primary antibodies: Rabbit anti-NGF (1:200 dilution), goat anti-GLUT1 (1:200 dilution) and goat anti-JNK (1:200 dilution), followed by an incubation with the corresponding horseradish peroxidase-conjugated secondary antibodies (1:3,000 dilution; GE Healthcare Life Sciences, Chalfont, UK), at room temperature for 2 h. All of the primary antibodies used for western blotting were purchased from Santa Cruz Biotechnology Inc. (Santa Cruz, CA, USA). Following several washes, the intensity of the visualization signal was detected using an Enhanced Chemiluminescence Substrate kit (Thermo Fisher Scientific Inc., Shanghai, China), and the relative protein levels were quantified using the Image J software system (National Institutes of Health, Bethesda, MD, USA). GAPDH served as an endogenous internal reference. The relative expression of each target protein was calculated as the ratio of the intensity of the target protein band as compared with GAPDH.

### Measurement of MVD

Any endothelial cell or endothelial cell cluster was considered a single countable microvessel, as described by previous methods (23). The tissues from the different groups were cut into 5-*μ*m serial sections, and immunohistochemical staining was used to detect factor VIII, in order to evaluate the MVD. Briefly, the rats were terminally anesthetized with isoflurane and the ascending aorta was perfused with saline, followed by 4% paraformaldehyde (Sigma-Aldrich) with 1.5% picric acid (Sigma-Aldrich) in 0.16 M phosphate buffer (pH 7.2–7.4). Following the perfusion, the muscle tissue around the incision site was harvested and post-fixed in the same fixative for 3–6 h, then replaced with 15% sucrose (Sigma-Aldrich) overnight. Muscle tissue sections (15 mm) were cut in a cryostat and processed for immunofluorescence. All of the sections were blocked with 5% donkey serum (Gibco-BRL, Carlsbad, CA, USA) in 0.3% Triton (Gibco-BRL) for 2 h at room temperature and incubated overnight at 4°C with factor VIII antibody (Santa Cruz Biotechnology Inc.). The sections were then incubated for 2 h at room temperature with Cy3-conjugated secondary antibody (1:300, Jackson ImmunoResearch Laboratories, Inc., West Grove, PA, USA). The stained sections were examined using a Leica fluorescence microscope (Leica Microsystems GmbH, Wetzlar, Germany), and the images were captured with a charge-coupled device Spot camera (Leica Microsystems GmbH). For the negative controls the primary antibodies were omitted. The factor VIII-positive sections were stained orange and were initially scanned at ×100 magnification. The three areas with the highest number of microvessels were selected, and were subsequently scanned at ×200 magnification. The mean number of microvessels was defined as the MVD (15).

### Statistical analyses

All statistical analyses were performed using SPSS version 17.0 (SPSS Inc., Chicago, IL, USA), and all data are expressed as the means ± standard deviation. Differences between the two groups were compared using a Student’s t-test and a one-way analysis of variance was used to compare the differences among ≥3 groups. Statistical diagrams were drawn using Excel (Microsoft Corporation, Redmond, WA, USA). A P<0.05 was considered to indicate a statistically significant difference.

## Results

### Changes in MWT values in response to SMIR and pinacidil administration

It has been previously reported that SMIR may induce mechanical allodynia in rats, but that it has no effects on thermal or cold hyperalgesia (14). As shown in Table I, the MWT was significantly decreased at each time point in the SMIR group, as compared with the control group, in a time-dependent manner. However, there were no significant differences in the MWT of rats from the sham operation group, comparing between before and after the sham surgery. Furthermore, pinacidil administration was shown to significantly attenuate the SMIR-induced reduction in MWT, in a dose-dependent manner. As compared with the control group, the MWT was significantly reduced in the low-dose pinacidil (10 *μ*g/kg) group 3, 7 and 12 days after the SMIR surgery. A significantly decreased MWT was also observed in the rats in the middle-dose pinacidil (25 *μ*g/kg) group 12 days after the SMIR surgery, as compared with the rats in the control group (P<0.05). Administration of high-dose pinacidil (50 *μ*g/kg) completely inhibited the SMIR-induced decrease in the MWT at all time points, 1,3, 7 and 12 days after the SMIR surgery.

These data indicate that the degree of mechanical allodynia was relative to the methods of operation, and that SMIR surgery could significantly increase the severity of mechanical allodynia, as compared to the incision alone. It was also shown that pinacidil could antagonize the SMIR-induced nociceptive response, restoring it to a normal level, likely through the activation of KATP channels.

### Changes in MVD around the incision site

**Figure 2:**
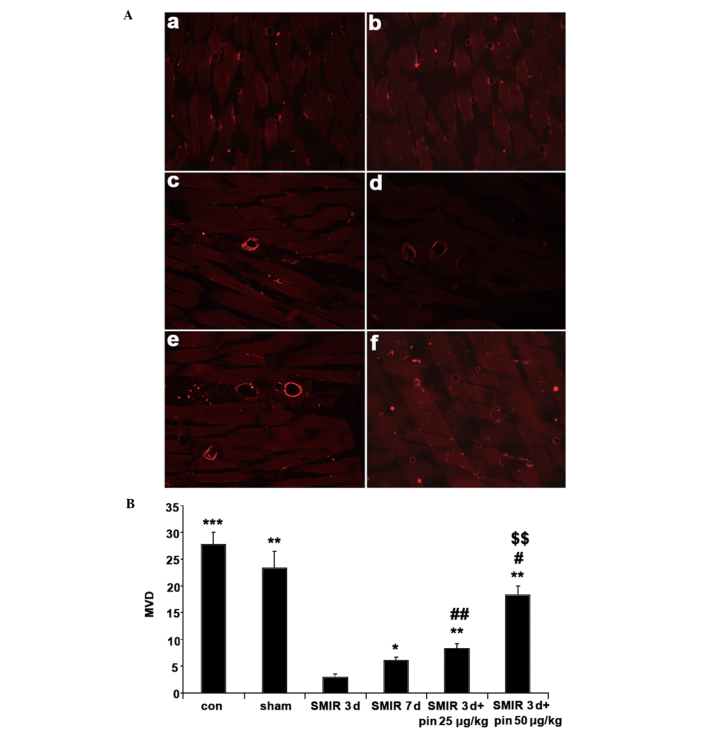
Comparison of the microvessel density (MVD) value in the groups of rats. (A) Immunohistochemical staining (magnification, x200) of factor VIII. The positive cells were stained orange. (a) Control group; (b) sham operation group; (c) SMIR group three days after surgery; (d) SMIR group seven days after surgery; (e) 25 *μ*g/kg pinacidil group; (f) 50 *μ*g/kg pinacidil group. (B) Quantitative analysis of MVD from immunohistochemical staining of factor VIII. *P<0.05, ^**^P<0.01, ^***^P<0.001 vs. SMIR group, three days after surgery; ^#^P<0.05, ^##^P<0.01 vs. control group; ^$$^P<0.01 vs. 25 *μ*g/kg pinacidil group. D, day; pin, pinacidil; con, control; sham, incision only operation.

As shown in Fig. 2, the values of MVD in the control and sham operation groups were high at the start of the experiment, or three days after the sham surgery, respectively, as compared with the MVD in the SMIR group. In the SMIR group, the MVD value was significantly increased seven days after the surgery as compared with three days after the surgery (P<0.05). Additionally, the MVD values in the middle-dose pinacidil and high-dose pinacidil groups were significantly higher as compared with the value in the SMIR group 3 days after surgery.

Furthermore, three days after surgery, the MVD values were shown to be significantly lower in the middle- and high-dose pinacidil groups, as compared with the control group, and the MVD value was significantly increased in the high-dose pinacidil group as compared with the middle-dose pinacidil group (P<0.01).

The results of the immunohistochemical staining suggested that the SMIR surgery caused an imbalance between apoptosis and proliferation of the vascular endothelial cells, resulting in the dynamic changes in MVD. It is therefore suggested that SMIR surgery may cause both microangiopathy and inflammation, and that SMIR surgery was able to decrease MVD significantly, as compared with incision alone. The present study also showed that the administration of pinacidil, prior to surgery, resulted in a dose-dependent increase in the MVD value and partly inhibited the SMIR-induced reduction in MVD. Pinacidil may improve the microenvironment around the incision site, thereby exerting its inhibitory effects on both peripheral and central sensitization.

### Changes in relative GLUT1 protein expression levels around the incision site

**Figure 3:**
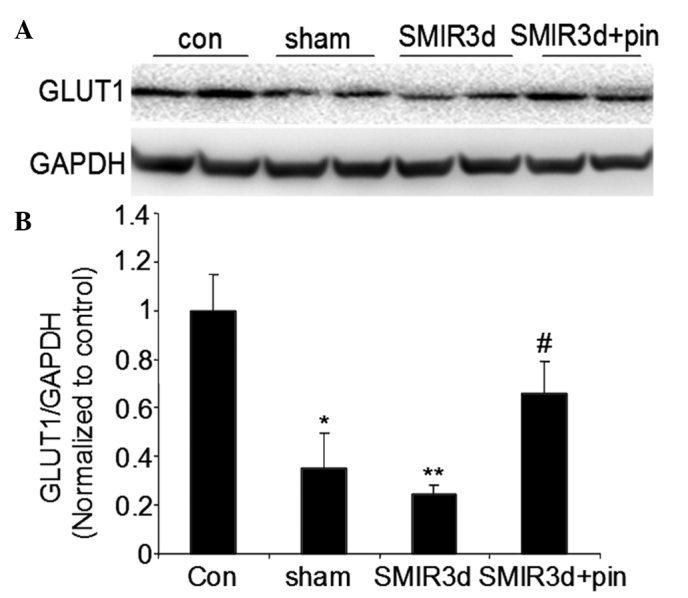
Comparison of glucose transporter-1 (GLUT1) protein expression levels in the different groups of rats. (A) Western blot analysis of GLUT1 protein expression levels in the different groups; (B) Quantification of GLUT1 protein expression levels, after normalization to GAPDH internal control. ^*^P<0.05, ^**^P<0.01 vs. control group; ^#^P<0.05 vs. SMIR group. SMIR, skin/muscle incision and retraction; d, day; pin, pinacidil; con, control; sham, incision only operation.

As compared with the GLUT1 protein expression level in the control group, the GLUT1 protein expression levels in the sham operation and SMIR groups were significantly reduced three days after surgery (P<0.05 and P<0.01, respectively). Furthermore, the GLUT1 protein expression level in the middle-dose pinacidil group three days after surgery was significantly higher, as compared with that in the SMIR group (P<0.05) (Fig. 3).

These data suggest that the SMIR procedure was able to significantly reduce the relative GLUT1 protein expression level, as compared with the incision alone. It was also shown that intraperitoneal administration of pinacidil prior to the SMIR surgery, could inhibit the SMIR-induced reduction in GLUT1 protein expression level and enhance local glucose utilization and basal metabolism. In addition, pinacidil could improve the microenvironment, and contribute to the inhibition of peripheral and central sensitization.

### Changes in relative NGF protein expression levels around the incision site

**Figure 4:**
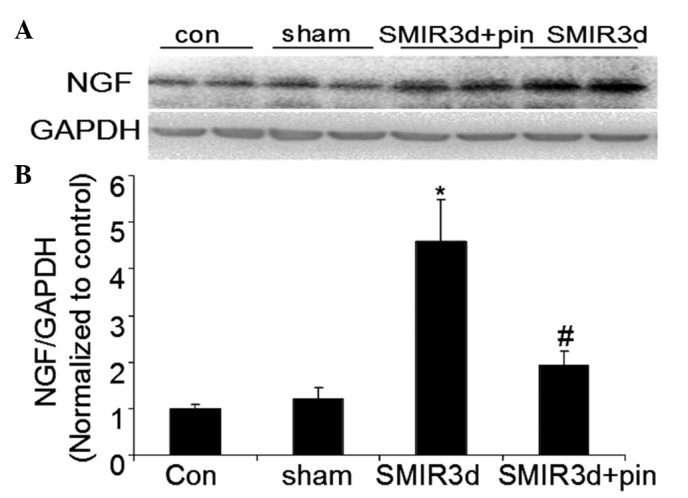
Comparison of nerve growth factor (NGF) protein expression levels in the different groups of rats. (A) Western blot of NGF protein expression levels in the different groups; (B) Quantification of NGF protein expression levels, after normalization to GAPDH internal control. ^*^P<0.01 vs. control group; ^#^P<0.05 vs. SMIR group. SMIR, skin/muscle incision and retraction; d, day; pin, pinacidil; con, control; sham, incision only operation.

In the SMIR group, the NGF protein expression level was significantly increased three days after surgery, as compared with the NGF protein expression level in the control group (P<0.01). There were no marked changes in the protein expression levels of NGF in the sham operation group after surgery. The NGF protein expression level in the middle-dose pinacidil group was markedly lower as compared with that in the SMIR group, three days after surgery (P<0.05) (Fig. 4).

These data indicate that the rats that underwent the SMIR surgery had a significantly higher relative NGF protein expression level, as compared with those treated with the incision alone. It was also shown that intraperitoneal administration of pinacidil prior to the SMIR surgery could inhibit the SMIR-induced increase in NGF protein expression level and improve the microenvironment, which may have an important contributory role in the inhibition of peripheral sensitization.

### Changes in relative spinal JNK protein expression levels around the incision site

**Figure 5:**
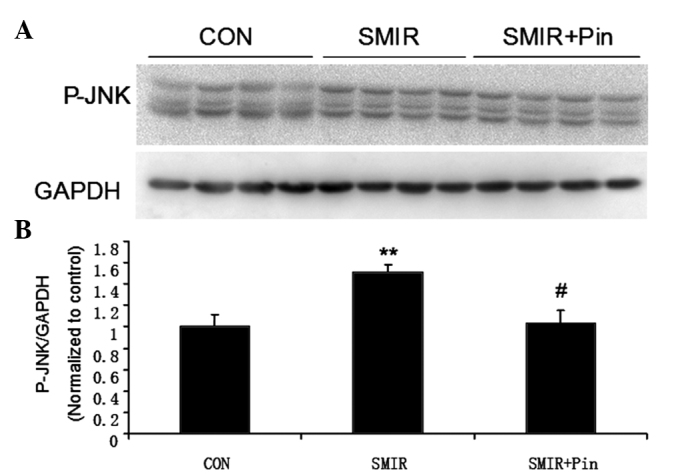
Comparison of peripheral C-jun N-terminal kinase (P-JNK) protein expression levels in the different groups of rats. (A) Western blot of P-JNK protein expression levels in the different groups; (B) Quantification of P-JNK protein expression levels, after normalization to GAPDH internal control. ^**^P<0.01 vs. control group; ^#^P<0.05 vs. SMIR group. SMIR, skin/muscle incision and retraction; pin, pinacidil; con, control; sham, incision only operation.

Following surgery, the p-JNK protein expression level was significantly increased in the SMIR group as compared with the protein expression level in the control group (P<0.01). Conversely, a significantly reduced level of p-JNK protein was found in the middle-dose pinacidil group, as compared with that in the SMIR group (P<0.05) (Fig. 5).

These data indicate that peripheral activation of KATP channels, prior to SMIR surgery, could suppress the SMIR-induced spinal JNK activation, and therefore inhibit central sensitization.

## Discussion

It has been previously reported that the formalin-induced animal model of inflammatory nociception could not simulate the microenvironment around the incision site (24,25). The present study suggests that during SMIR surgery, prolonged tissue retraction does not cause peripheral neuronal damage; thus, the SMIR model can provide a more accurate reflection of the microenvironment around an incision site. The results of the present study determined that three days after SMIR surgery, the MVD value was significantly decreased, as compared with the MVD value of the control group, whereas a marked increased MVD value was observed seven days after SMIR surgery. Therefore, it is hypothesized that various pathological conditions associated with an acute inflammatory response, including ischemia, anoxia and ischemia/reperfusion, may be present around the incision site in the SMIR model (26). Furthermore, the relative GLUT1 protein expression level in the SMIR group, three days after surgery, was significantly lower as compared with that in the control group, likely resulting in a reduction in glucose utilization and disorder of basal metabolism. In addition, a significant elevation in the relative NGF protein expression level was observed three days after surgery in the SMIR group as compared with the control group. An increased NGF protein expression level has previously been found to induce an inflammatory response and promote the release of algogenic substances (27–29). This would result in an increase to the sensitivity and excitability of primary nociceptive neurons, thereby resulting in peripheral sensitization following the SMIR procedure. A more severe presentation of mechanical allodynia was observed in the present study, three days after surgery in the SMIR group, as compared with one day after surgery. These findings indicate that SMIR surgery could induce peripheral and central sensitization, and that the inflammatory responses around the site of incision could promote excitation of the central nervous system, which is considered to be the developmental phase of both peripheral and central sensitization. The present study suggests that SMIR-induced inflammation of primary nociceptive neurons in the spinal cord could activate JNK and result in central sensitization, which may markedly increase the severity of mechanical allodynia. These data demonstrate that following SMIR surgery, the inflammatory responses around the incision site, which are induced by an imbalance between apoptosis and proliferation of vascular endothelial cells and disorder of basal metabolism, contribute to the development of peripheral and central sensitization. Vascular endothelial cell apoptosis is implicated as having a role in the generation of peripheral and central sensitization. Hence, it may be speculated that peripheral and central sensitization can be prevented by blocking the formation of the inflammatory microenvironment around the incision site.

In the present study, the rats in the sham group underwent a sham surgery involving a skin/muscle incision only. The results showed that although a reduction in the MVD value was noted in the sham operation group, there were no significant changes with regards to the MVD three days after the surgery. The observations also revealed that, as compared with the control group, the relative GLUT1 protein expression level in the sham operation group was significantly reduced three days after surgery; however, GLUT1 protein expression was further reduced in the SMIR group. Furthermore, in the sham operation group, no significant differences were observed in the relative protein expression of NGF as compared with the expression level prior to the surgery. These findings indicate that the microenvironment around the incision site was not capable of persistent excitation of nociceptors, resulting in peripheral and central sensitization. The rats that underwent a skin/muscle incision did not show obvious mechanical allodynia. MVD was therefore proven to be more favorable than GLUT1 for maintaining a balanced microenvironment. Collectively, the results from the present study confirm that the microenvironment around the incision site varied with the surgical methods used, and the retraction procedure was shown to be more likely to cause disorder of cell metabolism and deterioration of the microenvironment, including an increase in NGF protein expression and a reduction in GLUT-1 protein expression and MVD value. This resulted in peripheral and central sensitization, thereby increasing the severity of mechanical allodynia.

Previous studies have found that KATP channel activators can protect against endothelial cell dysfunction (30,31). Kir6.1/SUR2B is a subtype of the vascular KATP channels (32), and it has been suggested that SUR2 may be a therapeutic target of pinacidil (33). The results from the present study revealed that the administration of pinacidil increased the MVD value around the incision site, in a dose-dependent manner. It was also shown to partly inhibit the SMIR-induced reduction of MVD, resulting in a large increase in the low peripheral resistance to nutritional blood flow. Additionally, intraperitoneal administration of pinacidil prior to SMIR surgery inhibited the SMIR-induced reduction of GLUT1 protein expression levels, which can contribute to the maintenance of basal metabolism and glucose utilization. Pinacidil was also shown to exert an inhibitory effect on the SMIR-induced increase in NGF protein expression levels, and therefore prevent peripheral and central sensitization. These findings suggest that there were no inflammatory responses present in the primary sensory neurons around the incision site, due to inhibition of the formation of an inflammatory microenvironment by pinacidil. Furthermore, there were no marked changes in the spinal JNK protein expression levels in rats treated with pinacidil after surgery, indicating that pinacidil can inhibit central sensitization, and reduce the degree of mechanical allodynia in a dose-dependent manner. These findings suggest that persistent excitement of nociceptors was not elicited around the surgical site when KATP channels were activated prior to the activation of nociceptors. Thus, pinacidil can exert an inhibitory effect on peripheral and central sensitization.

In conclusion, the present study demonstrated that SMIR-evoked postoperative pain can be considered a combined process of microangiopathy and ischemia/reperfusion. It was also demonstrated that the microenvironment around the incision site can affect the development of peripheral and central sensitization. Furthermore, pinacidil demonstrated an inhibitory effect on the formation of the inflammatory microenvironment through activation of the KATP channels, thereby inhibiting peripheral and central sensitization. Pinacidil may be a potential treatment option in preemptive analgesia.

## Figures and Tables

**Table I:** Comparison of the mechanical withdrawal threshold in the control, sham, SMIR, and pinacidil groups of rats.

	Control (g)	Sham (g)	SMIR (g)	SMIR + pinacidil (g)		
				10 *μ*g/kg	25 *μ*g/kg	50 *μ*g/kg
Before SMIR surgery	23.1±1.3	23.4±1.3	23.0±1.4	24.3±1.2	23.1±1.3	23.34±1.34
After SMIR surgery						
1 day	23.7±1.3	22.0±0.3	18.4±1.7^a^	24.1±1.3	24.1±1.3	24.59±1.26
3 days	23.8±1.6	20.3±1.3^c^	10.9±1.6^b^	16.7±1.3^a^	24.5±1.3	23.54±1.15
7 days	23.4±1.2	22.1±1.2^c^	6.2±0.7^b^	19.0±1.0^a^	22.9±1.2	24.41±1.37
12 days	24.0±1.2	23.2±1.3^c^	8.3±0.9^b^	12.0±0.7^b^	15.4±0.9^a^	23.81±1.23

The data represent the means ± standard deviation.

a P<0.05,

b P<0.01 vs. control group;

c P<0.01 vs. SMIR group. SMIR, skin/muscle incision and retraction; g, grams.
